# Harmonization of three public health insurance schemes on emergency medical services

**DOI:** 10.1186/1471-2458-14-S1-O30

**Published:** 2014-01-29

**Authors:** Wannapha Bamrungkhet, Sutherada Chimnoi, Samrit Srithamrongsawat, Supasit Pannarunothai

**Affiliations:** 1 Faculty of Medicine, Naresuan University Phitsanulok 65000, Thailand; 2 Health Insurance System Research Office (HISRO), Nonthaburi 11000, Thailand

## Background

The study aimed to investigate the effects of harmonization of three public health insurance schemes on emergency patients.

## Materials and methods

The study used a telephone interview survey with semi-structured questionnaire. The population sample was selected by using simple random sampling with personal identification number of emergency patients who accessed health services under the policy during August-October 2012, and were also in the Emergency Claim online (EMCO) database. There were 292 emergency patients who were interviewed, which consisted of 105 cases of Universal Coverage Scheme, 95 cases of Social Security Scheme, and 92 cases of Civil Servant Medical Benefit Scheme.

## Results

Most of emergency patients were aware of the policy, but still misunderstood the definition of the emergency condition under the policy. Most of them accessed in-patient care. The majority (98%) accessed and selected health services by themselves, only 2% of patients called 1669 EMS to get access to hospital. Furthermore, 85% of patients reported that the priority reasons for selecting health services were the distance and quality standard of the hospitals. However, less than 17% of patients knew their benefit schemes and the referral process. Although most of patients did not want to be asked for the health insurance scheme before receiving health services, most of them were asked and had to pay cash at point of service then later reimbursed from the clearing house. This was a burden for the patients and their families. However, they did not know the hotline number where they can make complaints.

## Conclusions

Emergency patients were aware of the policy. However, being asked for health insurance scheme before receiving treatment was the practice still existed. Most of the patients accessed hospitals by themselves, which corresponded with the limited use of EMS system, and thus needed to be improved. However, the policy promoted that emergency patients could access to health services for free, but most of them had to pay at the point of service.

